# Directional postural sway tendencies and static balance among community-dwelling older adults with depression and without cognitive impairment

**DOI:** 10.1007/s40520-025-03144-y

**Published:** 2025-08-21

**Authors:** Jethro Raphael M. Suarez, Veronica B. Decker, Joon-Hyuk Park, Nichole R. Lighthall, Michael Joseph S. Dino, Ladda Thiamwong

**Affiliations:** 1 Department of Mechanical and Aerospace Engineering, University of Central Florida, Orlando, FL USA; 2 College of Nursing, University of Central Florida, Orlando, FL USA; 3 Disability, Aging, and Technology Cluster, University of Central Florida, Orlando, FL USA; 4 Department of Robotics and Mechatronics Engineering, Daegu Gyeongbuk Institute of Science and Technology, Daegu, Republic of Korea; 5 Department of Psychology, University of Central Florida, Orlando, FL USA

**Keywords:** Balance, Ellipse area, Mental health, BTrackS

## Abstract

**Background:**

Depression is prevalent among older adults and is known to negatively affect balance, ultimately leading to falls. However, few studies have investigated the effect of depression on static balance metrics beyond postural sway distance and area of older adults without mild cognitive impairment (MCI).

**Aims:**

To investigate if postural sway distance, sway area, medial-lateral (ML) sway range, anterior-posterior (AP) sway range, and center-of-pressure (COP) sway speed variability differed between non-cognitively impaired older adults with minimal-to-no depression (Minimally Depressive group) and mild-to-severe depression (Mildly-to-Severely Depressive group).

**Methods:**

A total of 204 community-dwelling older adults were included. Depression was measured using the Patient Health Questionnaire-9 (PHQ-9), MCI using the Rowland Universal Dementia Assessment Scale (RUDAS), and static balance metrics using the Balance Tracking System (BTrackS). Mann-Whitney U tests determined differences between groups.

**Results:**

Sway area, AP sway range, and ML sway range were significantly greater in the Mildly-to-Severely Depressive group than the Minimally Depressive group (*p* = 0.010, *p* = 0.016, and *p* = 0.031, respectively). Sway distance (*p* = 0.445) and COP sway speed variability (*p* = 0.193) were not significantly different between groups.

**Discussion:**

The findings revealed greater sway area, as well as greater ranges in the AP and ML directions, in the Mildly-to-Severely Depressive group when compared to the Minimally Depressive Group. Reduced concentration and affected postural stabilization mechanisms driven by depression may have attributed to these results.

**Conclusions:**

This study highlights the need for further understanding of how static balance metrics, such as directional sway, are affected by depression, thereby creating interventions tailored to individual’s postural sway characteristics to help reduce fall risk and improve balance.

**Trial registration:**

ClinicalTrials.gov (NCT05778604).

**Supplementary Information:**

The online version contains supplementary material available at 10.1007/s40520-025-03144-y.

## Introduction

Depression is a common mood disorder among older adults and is typically associated with reduced energy levels, loss of confidence, impaired concentration, slow movement, and reduced social activity [1–6]. Although primarily considered a mood disorder, depression affects the physical body as much as the mental state. It poses a serious health concern among older adults due to its strong association with falls, fear of falling (FOF), and reduced cognitive function [7, 8], as well as decreased balance, physical function, and motor performance [9]. An aversion to physical activity can lead to muscle weakness and poor balance, which results in a higher risk of falling or actual falls [10]. Intervention programs are continuously being developed and implemented to prevent older adults from falling by aiming to improve their social and physical capabilities [11, 12]. However, to create effective intervention programs, it is essential to understand how confounding factors, such as depression, affect older adults’ risk and incidence of falls. The effect of depression on the physical function of older adults is a complex bidirectional cycle and is not yet fully understood. Therefore, the investigation of the physical function of older adults with depression, particularly their balance performance, compared with those who are not depressed has become a popular topic in the field of geriatrics.

A review of physical function assessments for individuals with depression found that a multitude of studies have assessed the static and dynamic balance of individuals with depression through physical function tests and electronic measurement devices [13]. While studies have been conducted for both young and older adults, a focus on older adults is warranted due to their higher risk of falling [14]. Collectively, studies that investigated the balance of older adults with depression found that they have significantly worse balance than those without depression, as evidenced by increased postural sway and higher scores from performance-based measures such as the Berg Balance Scale [9, 15–17]. To date, in studies exploring both static and dynamic balance of older adults with depression, a more prominent focus has been on dynamic balance. While dynamic balance is more complex, a deeper understanding of older adults’ static balance performance is essential, as it is the foundation of balance control.

To accurately assess the impact of depression alone on the static balance of older adults, it is essential to recognize other conditions that are commonly associated with depression. Mild cognitive impairment (MCI) is a condition that typically coexists alongside depression [18], and it has been established that older adults with MCI have significantly worse balance than those without [19–21]. Although MCI and depression can exist simultaneously, instances of one or the other could occur for individuals, and therefore, an understanding of how each factor affects an individual is necessary. It has been previously found that the presence of depressive symptoms in older adults with MCI negatively affected their balance when compared to older adults with MCI and without depressive symptoms [16]. Prior studies have also focused on the effect of depression alone on the balance of older adults, specifically those without MCI, and found that those with increased depression have worse balance than those without depression, as seen through increased sway distance and area [9, 22]. However, metrics beyond sway distance and area have rarely been explored regarding older adults with depression and without MCI.

Laboratory-grade force plates, suggested to be the gold standard for measuring balance performance [23], have been previously used to assess the static balance of older adults with and without depression [19, 20, 22, 24–26]. Force plates allow for in-depth analysis of an individual’s static balance performance by calculating balance metrics such as sway distance and sway area. Sway distance can be defined as the total distance that an individual’s center-of-pressure (COP) travels while swaying during a static stance, whereas sway area can be defined as the elliptical shape that encloses the COP distance traveled during a static stance. Though commonly used, ambiguity exists when considering these two metrics alone. Increased sway distance could signify either one large area or multiple small areas of COP movement. Incorporating sway area with sway distance aids in providing more information regarding the COP movement of an individual, but a level of ambiguity still exists. To reduce ambiguity, metrics beyond these two should be considered. As mentioned previously, balance metrics beyond sway area and distance have rarely been considered when exploring the balance performance of older adults with depression and without MCI. Postural sway distance and area together can provide the severity of postural sway during static standing, but they do not provide information regarding speed of sway or directional movement, specifically in the anterior-posterior (AP) and medial-lateral (ML) directions. Such information can reveal postural control dysfunction and musculoskeletal weaknesses, thereby providing information that could be used to create tailored interventions that aim to reduce fall risk and improve balance. With this in mind, an investigation was warranted to explore the directional tendencies of older adults with depression and without MCI.

To further understand how depression alone affects the static balance of older adults independent of MCI, this study aimed to compare the sway distance, sway area, ML sway range, AP sway range, and COP sway speed variability between older adults without MCI who exhibit minimal-to-no depression and those who exhibit mild-to-severe depression. It is hypothesized that older adults with mild-to-severe depression will exhibit increased sway distance and area, which aligns with the results from previous studies [9, 16, 22], but more specifically, ranges of sway for older adults with mild-to-severe depression will be more extensive in both the ML and AP directions. Furthemore, COP sway speed variability in older adults with mild-to-severe depression is hypothesized to be higher than those with minimal-to-no depression, indicative of less-effective postural control [27].

## Methods

This cross-sectional investigation was part of a larger study, federally funded by the National Institute on Minority Health and Health Disparities (R01MD018025), and pre-registered on ClinicalTrials.gov (NCT05778604). The protocol for the study was approved by the University of Central Florida Institutional Review Board (STUDY00003206), adhered to the Declaration of Helsinki, and has been previously published elsewhere [28]. The data for this study was securely stored and managed using Research Electronic Data Capture (REDCap), a secure, web-based application designed to store data for research studies [29, 30].

### Setting and sample

The study was conducted in the Greater Orlando, FL, USA metropolitan area and recruitment was accomplished through word-of-mouth, flyer distribution, and partnership with local communities. A total of 319 community-dwelling older adults were recruited. Participants were included in this study if they were at least 60 years of age, could stand on their own, were low-income status based on the 2019 poverty thresholds relative to family size [31], completed the Rowland Universal Dementia Assessment Scale (RUDAS), completed the Patient Health Questionnaire-9 (PHQ-9), and fully completed a balance assessment on the Balance Tracking System (BTrackS). Participants who were unable to stand unassisted for at least 2 min (maximum duration of the static balance assessment utilized in this study), receiving treatment from any form of rehabilitation facility (e.g., acute, subacute, long-term care settings), or received a score of 22 or lower on the RUDAS (signifying MCI) were excluded. After screening for inclusion/exclusion criteria, 204 participants were included in this study. All participants provided written informed consent before participation.

### Demographic measurements

Demographic information (i.e., age, sex, and general health) was obtained using a self-reported survey. Height and weight were assessed without shoes using a digital physician scale with a built-in stadiometer (Health-O-Meter^™^, Model 402KL, McCook, IL, USA).

### Cognitive screening

The RUDAS was used to identify MCI by assessing body orientation, praxis, drawing, judgment, memory, and language for the study participants. Body orientation was assessed by identifying various body parts, praxis by alternating hand movements, drawing by the recreation of a cube on paper, judgment by the answers given to a road-crossing scenario, memory by recalling a 4-item list, and language by reciting eight animals in under one minute [32, 33]. The total score of the RUDAS ranges from 0 to 30, with a score of 22 or less indicating the possibility of MCI and dementia [34]. The RUDAS has been reported to have a sensitivity and specificity of 89% and 98%, respectively, as well as inter-rater and test-retest reliabilities ranging from 0.71 to 0.99 and 0.86–0.98 [32, 33, 35, 36].

### Depression screening

The Patient Health Questionnaire-9 (PHQ-9) is a nine-item instrument that measures the severity of depression based on self-reported answers and has accurately identified depression among older adults [37]. The questions on this instrument utilize a 4-point Likert scale based on how often an individual has been bothered by specific problems. Answers range from 0 (“Not At All”) to 3 (“Nearly Every Day”). Examples of the prompts presented are “Little interest or pleasure in doing things” and “Feeling down, depressed, or hopeless”. The PHQ-9 has a high internal consistency (Cronbach α = 0.89) and has shown high sensitivity and specificity (88%) in detecting major depression for individuals with a PHQ-9 score ≥ 10 [37–40]. The PHQ-9 typically utilizes a five-group classification based on the total score of an individual (minimal depression, mild depression, moderate depression, moderately severe depression, and severe depression) [39]. However, for the purposes of this study, a two-group classification was utilized for comparative group analysis to achieve more balanced group sizes among the total sample size: a “Minimally Depressive group” (PHQ-9 score ≤ 4) and a “Mildly-to-Severely Depressive group” (PHQ-9 score ≥ 5).

### Static balance assessment

The Balance and Fall Risk Protocol from the BTrackS was utilized for static balance assessment and administered via the BTrackS Assess Balance software (Version 7.5.5, Balance Tracking Systems Inc., San Diego, CA, USA) interfaced on a Microsoft Windows computer (Version 10, Microsoft Corporation, Redmond, WA, USA). The BTrackS Balance and Fall Risk Protocol involves participants performing four 20-second standing trials with feet shoulder-width apart, hands on hips, head facing forward, and eyes closed. The first trial is a practice trial, whereas the following three trials are averaged to calculate COP metrics. The balance metrics considered for this study were COP path length (representative of sway distance), 95% Elliptical Sway Area (95% ELL; representative of sway area), range of ML sway (RG-ML), range of AP sway (RG-AP) and COP sway speed variability. 95% ELL is the smallest ellipse that fits 95% of the COP path created by the participant (cm^2^), RG-ML is the total range that the participant’s COP traveled in the medial and lateral direction (cm), RG-AP is the total range that the participant’s COP traveled in the anterior and posterior direction (cm), and COP sway speed variability is the standard deviation of instantaneous sway speed (cm/s). The BTrackS Balance and Fall Risk Protocol has been found to have an excellent intraclass correlation coefficient (ICC = 0.83) and has been used in previous studies regarding older adults [26, 41].

### Statistical analysis

**Fig. 1:**
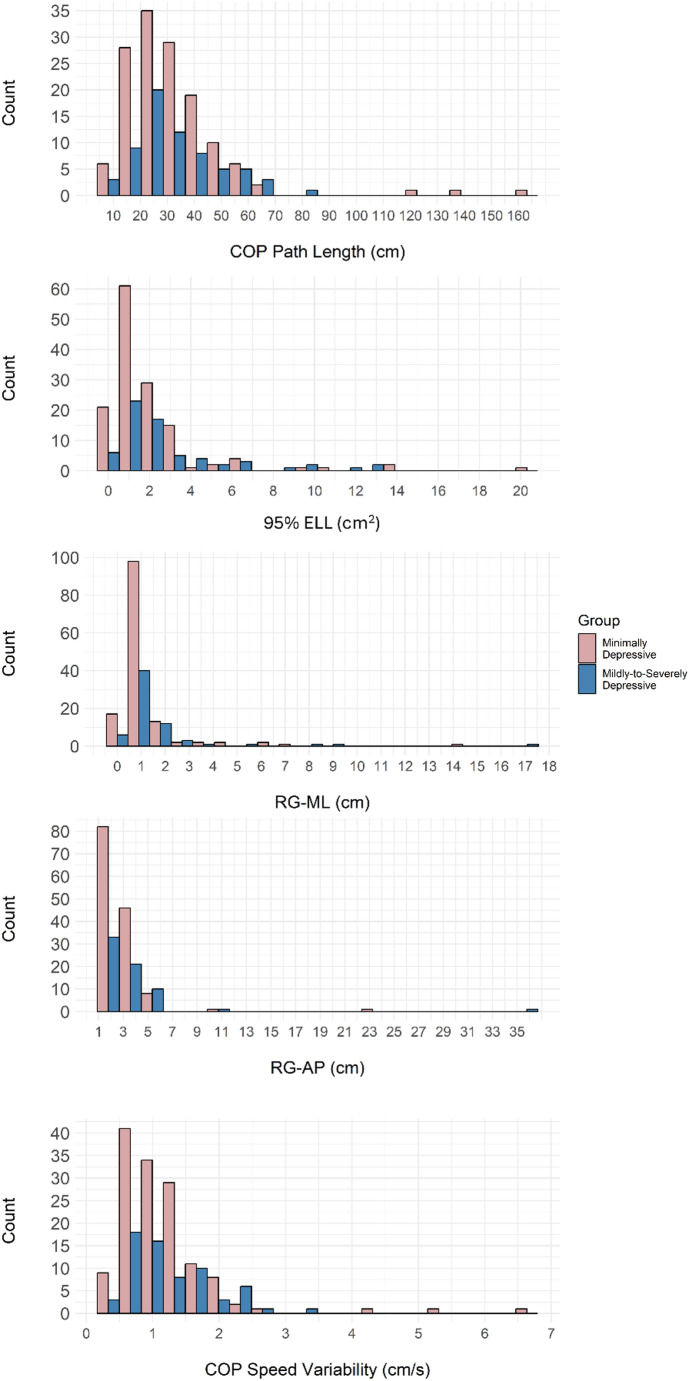
Distribution of static balance metrics by group

RStudio (Version 4.3.1) was used for statistical analyses. All static balance metrics (COP path length, 95% ELL, RG-ML, RG-AP, and COP sway speed variability) were compared between the Minimally Depressive and Mildly-to-Severely Depressive group. Anderson-Darling tests found that all metrics were nonnormally distributed (results presented in Supplementary Table 1). The distribution of all metrics included in this investigation has been presented in Fig. 1. Mann Whitney-U tests were used to determine any statistical differences among all considered static balance metrics between the two groups.

## Results

**Fig. 2:**
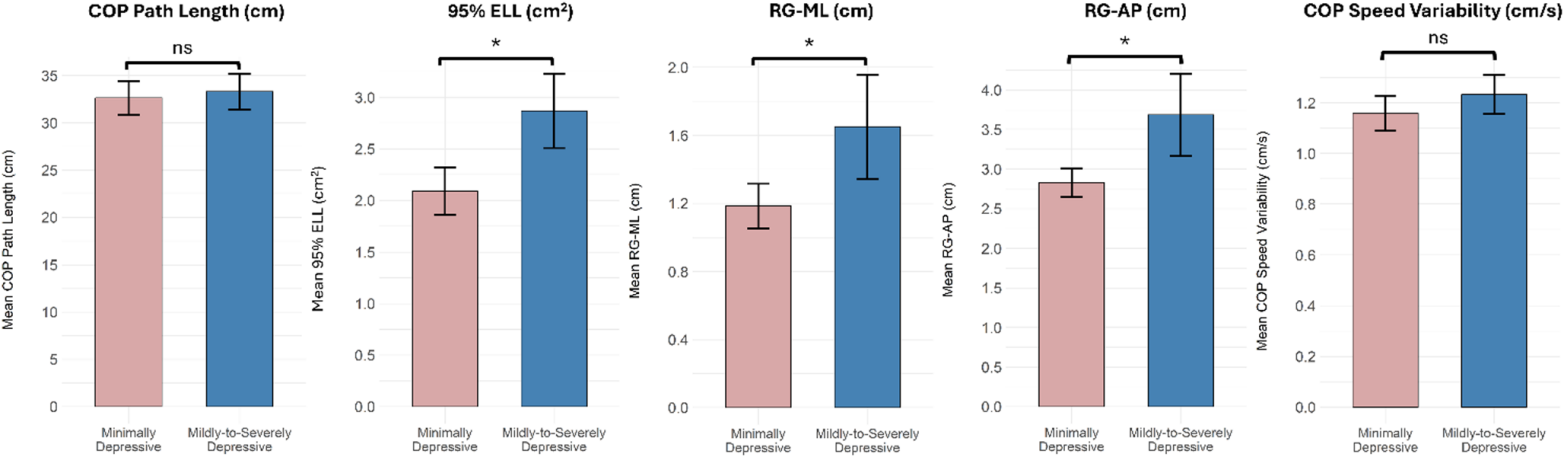
Comparison of static balance metric means by group

A total of 204 community-dwelling older adults were included in this study, with a mean age of 74 ± 7.3 years and a majority being female. Demographics per group, as well as for the overall population of the study, are presented in Table 1. Nonsignificant differences were found between groups regarding age (*p* = 0.890) and BMI (*p* = 0.373). A greater number of older adults were categorized into the Minimally Depressive group (68%) than the Mildly-to-Severely Depressive group. Mann-Whitney U tests showed that there were no significant differences for COP path length (*p* = 0.445) and COP sway speed variability (*p* = 0.193) between the Minimally Depressive group and Mildly-to-Severely Depressive group, but significant differences between the two groups were found for 95% ELL (*p* = 0.010), RG-ML (*p* = 0.031), and RG-AP (*p* = 0.016). Small to moderate effect sizes were found among the balance metrics that significantly differed between the two groups (r_95% ELL_ = 0.182; r_RG−ML_ = 0.151; r_RG−AP_ = 0.169). All results from the Mann-Whitney U tests are presented in Table 2, whereas the means and standard errors for each static balance metric across the two groups are visually presented in Fig. 2.

**Table 1:** Sample characteristics by depressive groups

Depressive Group	Minimally Depressive (*N* = 138)	Mildly-to-Severely Depressive (*N* = 66)	Total (*N* = 204)
**Sex**, distribution			
Female	122 (88%)	56 (85%)	178 (87%)
Male	16 (12%)	10 (15%)	26 (13%)
**Age (years)**, mean ± SD	74.1 ± 7.2	73.8 ± 7.6	74.0 ± 7.3
**Age (years)**, range	61.1–89.0	60.9–92.0	60.9–92.0
**BMI (kg/m**^**2**^**)**, mean ± SD	30.1 ± 5.7	31.6 ± 8.1	30.6 ± 6.6
**General Health**, distribution			
Poor	0 (0%)	3 (5%)	3 (1%)
Fair	13 (9%)	22 (33%)	35 (17%)
Good	69 (50%)	24 (36%)	93 (46%)
Very good	49 (36%)	16 (24%)	65 (32%)
Excellent	7 (5%)	1 (2%)	8 (4%)
**Education**, distribution			
Lower than high school	13 (9%)	11 (17%)	24 (12%)
High school	60 (44%)	28 (42%)	88 (43%)
College or above	65 (47%)	27 (41%)	92 (45%)
**Financial Status**, distribution			
Much less than adequate	6 (4%)	9 (14%)	15 (7%)
Less than adequate	19 (14%)	14 (20%)	33 (16%)
Just enough	79 (57%)	33 (50%)	112 (55%)
More than enough	30 (22%)	9 (14%)	39 (19%)
Much more than enough	4 (3%)	1 (2%)	5 (3%)
**Living Status**, distribution			
Alone	75 (55%)	31 (47%)	106 (52%)
With partner/spouse	35 (25%)	15 (23%)	50 (24%)
With family/friend	22 (16%)	16 (24%)	38 (19%)
Other	6 (4%)	4 (6%)	10 (5%)
**PHQ-9 Score**, mean ± SD	1.3 ± 1.4	9.3 ± 4.2	3.9 ± 4.6

*BMI* body mass index; *PHQ-9* Patient Health Questionnaire-9

**Table 2:** Static balance metrics and Mann-Whitney U test results

Variable	Minimally Depressive (*N* = 138)	Mildly-to-Severely Depressive (*N* = 66)	*p* values	U values	Effect Sizes (*r*)
**COP path length (cm)**, mean ± SD	32.6 ± 20.9	33.3 ± 15.5	0.445	4252.5	0.053
**95% ELL (cm**^**2**^**)**, mean ± SD	2.1 ± 2.7	2.9 ± 2.9	0.010	3530.5	0.182
**RG-ML (cm)**, mean ± SD	1.2 ± 1.5	1.7 ± 2.5	0.031	3703.5	0.151
**RG-AP (cm)**, mean ± SD	2.8 ± 2.1	3.7 ± 4.2	0.016	3603.0	0.169
**COP sway speed variability (cm/s)**, mean ± SD	1.2 ± 0.8	1.2 ± 0.6	0.193	4040.0	0.091

*COP* center of pressure; *95% ELL* 95% elliptical area; *RG-ML* range of medial-lateral sway; *RG-AP* range of anterior-posterior sway

## Discussion

The purpose of this study was to compare static balance metrics, specifically postural sway distance, sway area, AP sway range, ML sway range, and COP sway speed variability, between older adults with no MCI who exhibit minimal-to-no depression (Minimally Depressive group) and those who exhibit mild-to-severe depression (Mildly-to-Severely Depressive group). The results indicated that sway area (95% ELL), AP sway range (RG-AP), and ML sway range (RG-ML) significantly differed between the two groups, such that the Mildly-to-Severely Depressive group showed greater values in comparison to the Minimally Depressive group. These findings supported the original hypotheses. In contrast, sway distance (COP path length) and COP sway speed variability were not significantly different between groups, contrary to expectations.

### Comparison with previous studies

Two studies were found to have assessed the static balance of older adults with depression and no cognitive impairment and reported increased sway distance and area in depressed older adults [9, 22]. However, the procedures for each differed from the current study. One study involved participants standing on a force plate with arms to the side, feet apart, and eyes open during two separate tasks: standing still for 30 s (single task) and standing still while reciting items from memory (dual task) [9]. For direct comparison to the current study, only sway area and sway distance during the single tasks were considered. Although visual conditions differed, sway distance was expected to align with the results of the mentioned study due to the previously found conclusion that visual conditions (eyes open or closed) can be disregarded during static balance tests [42]. Regardless, it is likely that the mismatch in results regarding sway distance from the mentioned study and the current study might have been due to inconsistent durations of standing trials between them. The sway distance from the mentioned study was the average sway distance over three 30-second standing trials, whereas the current study utilized the average sway distance over three 20-second standing trials. Furthermore, the mentioned study did not indicate a practice trial, whereas the BTrackS assessment used in this study includes a practice trial. The shorter duration of trials, as well as the existence of a practice trial, could account for the insignificant difference in sway distance for the current study.

The second related study involved participants standing in five standing positions for 10 s with eyes closed while balance was measured using an application on a mobile phone [22]. Although the mentioned study and the current study both involve eyes closed conditions, likely, a significant difference in sway distance between groups was not found likely due to the BTrackS assessment utilizing a singular stance. Stances that involve reducing the base of support (e.g., tandem stance) challenge the balance of an individual, which would naturally result in increased postural sway.

For the present study, greater sway in the AP and ML directions was found in the Mildly-to-Severely Depressive group. The greater difference in such ranges could be attributed to several factors. Depression is associated with decreased physical activity and muscle weakness/fatigue [1, 43, 44]. During AP stabilization, an ankle strategy is utilized to maintain balance and includes the use of the plantarflexors and dorsiflexors of the ankle (e.g., tibialis anterior and posterior, extensor digitorum longus, and hallucis longus), whereas ML stabilization requires the use of hip abductors/adductors, along with the invertors/evertors of the ankle, to maintain balance [45]. Since depression is negatively associated with physical activity in older adults, the absence or reduced amount of physical activity could lead to overall weakness in such muscles and may be a physiological reason for difficulty in maintaining AP and ML stabilization. Additionally, depression can impair focus and attention, which may lead to the greater sway ranges due to reduced concentration. Although both directions of sway range significantly differed between groups, there was a greater difference in sway range mean for the AP direction in comparison to the ML direction. A potential factor attributing to this is the stance used in the balance assessment. The BTrackS assessment requires participants to place their feet shoulder-width apart. This stance naturally provides a wider base of support in the ML direction. Therefore, this posture facilitates inherently better stability in the ML direction, thereby possibly attributing to the greater difference in AP movement when compared to ML movement. Different stances may reveal greater difference between groups for ML sway range.

Additionally, it was hypothesized that COP sway speed variability would be significantly greater in the Mildly-to-Severely Depressive group due to previous work finding that greater COP sway speed metrics were exhibited in depressed individuals in comparison to healthy individuals [27]. However, in the present study, COP sway speed variability did not signicantly differ between groups. Similar to the restriction in the ML direction, it is possible that the stance/task utilized by the BTrackS Fall Balance and Fall Risk Protocol may not effectively challenge/test COP sway speed due to having a wide base of support. Previous work has incorporated tasks in addition to static standing (e.g., counting backwards), as well as different stances, which could facilitate more movement during static standing [27].

### Relationship with the brain

Depression has been previously found to affect the basal ganglia, through the presence of lesions [46]. The basal ganglia are structures in the brain that help regulate mechanisms involved in postural control, such as balance-correcting responses during quiet stance [47]. Due to the functional role of the basal ganglia, individuals with lesions on the basal ganglia have been found to have impaired balance control [48]. This relationship has previously been suggested to be the link between depression and impaired balance and could very well be a neurological reason for the findings of this study. Specifically, the findings of this study may suggest that lesions on the basal ganglia caused by depression could also suppress the mechanisms that control AP and ML stabilization.

### Clinical implications

To our knowledge, this is the first study to focus on measures of static balance beyond postural sway distance and area alone (i.e., directional ranges and COP sway speed variability) of older adults without MCI and with depression. While prior work found that older adults with depression exhibit greater postural sway [16], the findings of this study specifically reveal that older adults with depression and without MCI have significantly greater sway in both the AP and ML directions. This information may aid in the creation/improvement of tailored invervention programs aimed towards improving the balance of older adults with depression, especially for the prevention of falls. It has been previously found that the second-most effective approach towards reducing falls in older adults is the implementation of an exercise component, with the most effective approach being a culmination of different approaches (e.g., exercise classes, biofeedback, educational pamphlets) [49]. When developing exercise regimes for fall prevention programs or balance training interventions tailored towards older adults with depression, clincians and researchers can incorporate exercises that work to strengthen the muscles that are specifically used for both AP and ML stabilization (plantarflexors/dorsiflexors of the ankle, hip abductors/adductors, etc.).

### Limitations and future directions

Although established criteria for categorizing depression levels using the PHQ-9 exist [38], this study combined different levels of depression to create two distinct groups based on the distribution of older adults within each depression level (Minimally Depressive and Mildly-to-Severely Depressive Group). The categorization method presented in this study was chosen to create two groups that distinctly differed in levels of depression while attempting to maintain close-to-equal sample sizes between the two groups. The insignificant results of this study, specifically in the postural sway distance and COP sway speed variability metrics, could be attributed to the uneven sample sizes between the two groups. It is possible that statistical significance could be achieved in such metrics through the addition of older adults who exhibit mild-to-severe depression.

As previously mentioned, the BTrackS protocol requires individuals to stand with their feet shoulder-width apart. This stance naturally provides a wider base of support in the ML direction and facilitates easier movement in the AP direction than in the ML direction. Additionally, the BTrackS protocol only utilizes an “eyes-closed” condition, which was originally selected to isolate the proprioceptive and vestibular functions. However, this may be difficult for individuals who are visuo-dependent. The inclusion of a variety of stances, specifically stances that minimize the base of support (e.g., feet together, tandem), as well as an “eyes-open task”, could be considered to determine if older adults with depression truly move in the AP direction more than the ML direction during static standing, as well have increased COP sway speed variability.

A variety of metrics are known to affect the balance of individuals, such as age, BMI, physical activity levels, and medication use. The inclusion of age and BMI as covariates were originally considered for this study. However, comparative analyses were conducted, and no significant differences in age and BMI between the Minimally Depressive and Mildly-to-Severely Depressive groups were found; hence, it was not included in this study. Regarding physical activity measurements, while the parent study utilized wrist-worn accelerometers to calculate participants’ physical activity levels, compliance regarding the wear time of the devices was low, which would have led to a significant decrease in the sample size of this current study. Futhermore, the parent study utilizes self-reported demographic surveys that include a question regarding the use of medication to assist with sleeping, but no information regarding medication related to depression. Therefore, such information is unknown. Inclusion of such information (through ANCOVA or regression analysis) could aid in future analysis regarding static balance performance of older adults without MCI and with depression.

Additionally, the environment in which the balance tests were administered was not always consistent. Due to the nature of the study, balance tests were conducted at neighborhood senior living and community centers across the Central Florida area for the ease of the participants. Although the tests were administered similarly at all research sites, environmental factors such as noise could not be controlled, which could have possibly affected the performance of the participants in this study. Although significant differences were found between the Minimally Depressive and Mildly-to-Severely Depressive group for sway area, AP sway range, and ML sway range, the cross-sectional nature of this study limits the ability to draw causal inferences and therefore, the findings should be interpreted as associative and not causal. Longitudinal or experimental studies are warranted to fully determine causality. Collectively, these factors may have influenced the overall results. Future research should aim to address such factors (increase sample size, include covariates to aid in analysis, and explore the use of different balance stances) to fully determine the effects of depression on the static balance performance of older adults without MCI.

## Conclusions

Depression is prevalent among older adults and is known to affect physical function, particularly balance. Thorough static balance assessments are necessary to fully understand the stationary capabilities of older adults. The results from this study show that community-dwelling older adults with no MCI and with mild-to-severe depression have significantly greater sway area and sway range in the AP and ML direction than those with minimal-to-no depression. However, significant differences in sway distance and COP sway speed variability were not found, possibly due to the nature of the static balance assessment utilized for this study (eyes-closed, feet shoulder-width apart). Overall, considering balance metrics beyond sway distance and area revealed differences in directional ranges that would otherwise not have been found if focusing on sway distance and area alone. These findings may aid in the creation and improvement of tailored intervention strategies and assistive devices for improving the balance of older adults with depression. Future work regarding the analysis of such static balance metrics during multiple stances, during an eyes open condition, and with the inclusion of covariants such as physical activity levels and medication use is warranted to fully determine how depression affects the static balance performance of older adults without MCI.

## Electronic supplementary material

Below is the link to the electronic supplementary material.

Supplementary Material 1

## Data Availability

The data that supports the results of this study are available from the corresponding author upon reasonable request.
