# Supplementary material for: Directional postural sway tendencies and static balance among community-dwelling older adults with depression and without cognitive impairment
Source: Aging Clin Exp Res. 2025 Aug 21;37(1):255. doi: 10.1007/s40520-025-03144-y (PMC12370788; doi:10.1007/s40520-025-03144-y)
Supplement: Supplementary file 1 — Supplementary Material 1 [file 40520_2025_3144_MOESM1_ESM.docx]

**Supplementary Table 1** Results from Anderson-Darling normality tests between groups.

| **Variable** | **Minimally**  **Depressive**  **(*N* = 138)** | **Mildly-to-Severely Depressive**  **(*N* = 66)** |
| --- | --- | --- |
| **COP path length (cm)** | *p* < 0.001 | *p* < 0.001 |
| **95% ELL (cm^2^)** | *p* < 0.001 | *p* < 0.001 |
| **RG-ML (cm)** | *p* < 0.001 | *p* < 0.001 |
| **RG-AP (cm)** | *p* < 0.001 | *p* < 0.001 |
| **COP sway speed variability (cm/s)** | *p* < 0.001 | *p* < 0.001 |

*COP* center of pressure; *95% ELL* 95% elliptical area; *RG-ML* range of medial-lateral sway; *RG-AP* range of anterior-posterior sway.
